# RESILIENCE AND RECOVERY IN OLDER ADULTS: A COMPLEX SYSTEMS APPROACH

**DOI:** 10.1093/geroni/igz038.1380

**Published:** 2019-11-08

**Authors:** Jerrald Rector, Jerrald Rector, Marcel G Olde Rikkert, René Melis

**Affiliations:** 1 Radboud University medical center, Nijmegen, Netherlands; 2 Radboud university medical center, Nijmegen, Gelderland, Netherlands

## Abstract

In medicine, we still cannot objectively assess who will recover from health stressors imposed by disease or its treatment. If resilience, the dynamic ability to respond to and recover from health stressors, is considered as an emergent feature of a complex system, then methodology from complexity science may help us quantify the health-promoting features that support the recovery process. This presentation describes ongoing work aimed at empirically testing the concept and predictive value of resilience by examining the extent to which small-scale responses of bodily systems (i.e. heart rate, activity) to natural (micro)perturbations and indicators of loss of complexity are related to physical functioning and recovery throughout the journey of 120 geriatric inpatients. Dynamic indicators of resilience (variance, autocorrelation) and multiscale entropy measures were estimated from continuous heart rate and accelerometer data and compared to measures of patients’ physical functioning (e.g., ADLs, frailty) at admission, discharge and 3 months later.

